# Exopeptidase combination enhances the degradation of isotopically labelled gluten immunogenic peptides in humans

**DOI:** 10.3389/fimmu.2024.1425982

**Published:** 2024-10-16

**Authors:** Sulayman Mourabit, Sarah Römer, Erin R. Bonner, Fabian Winter, Julian Tschollar, Mladen V. Tzvetkov, Werner Weitschies, Stefan Engeli, Werner Tschollar

**Affiliations:** ^1^ AMYRA Biotech AG, Basel, Switzerland; ^2^ Department of General Pharmacology, Institute of Pharmacology Center of Drug Absorption and Transport (C_DAT), University Medicine Greifswald, Greifswald, Germany; ^3^ Department of Pharmaceutical Technology and Biopharmaceutics, Institute of Pharmacy, Center of Drug Absorption and Transport (C_DAT), University of Greifswald, Greifswald, Germany; ^4^ Department of Clinical Pharmacology, Institute of Pharmacology, Center of Drug Absorption and Transport (C_DAT), University Medicine Greifswald, Greifswald, Germany

**Keywords:** gluten, gluten immunogenic peptide, celiac disease, exopeptidase, glutenase, 33-mer, enzyme therapy, enzyme supplementation

## Abstract

**Introduction:**

Celiac disease is a common autoimmune-like enteropathy caused by an aberrant response to incompletely digested dietary gluten. Gluten immunogenic peptides including the immunodominant 33-mer are thought to be resistant to proteolytic digestion by human gastrointestinal peptidases. We developed a novel enzyme therapy approach to support gluten peptide digestion using a combination of two tandem-acting exopeptidases, AMYNOPEP, that complement the intrinsic enzymatic activity of intestinal brush border enterocytes.

**Methods:**

We evaluated the effects of AMYNOPEP supplementation on 33-mer degradation *in vitro* and *in vivo.* In a cross-over clinical study, healthy volunteers with no gastrointestinal disorders were given stable isotope (SI) labelled 33-mer peptides in the presence of varying peptide substrates and caloric loads, with and without AMYNOPEP. 33-mer degradation products (SI-labelled single amino acids) were measured in the blood plasma using LC-MS/MS.

**Results:**

AMYNOPEP achieved rapid, complete amino-to-carboxyl terminal degradation of the 33-mer *in vitro*, generating single amino acids and dipeptides. In healthy volunteers, AMYNOPEP supplementation significantly increased 33-mer degradation and absorption of SI-labelled amino acids even in the presence of competing substrates. Specifically, we observed a 2.8-fold increase in the C_max_ of stable isotope-labelled amino acids in the presence of wheat gluten. The absorption kinetics of labelled amino acids derived from 33-mer digestion with AMYNOPEP closely resembled that of SI-labelled X-Proline dipeptides administered without enzyme supplementation, highlighting the rapid hydrolytic activity of AMYNOPEP on polypeptides.

**Conclusions:**

AMYNOPEP achieved complete degradation of the 33-mer into single amino acids and dipeptides *in vitro* and significantly improved 33-mer degradation kinetics in healthy volunteers, as measured by labelled amino acid detection, warranting further investigation into the potential therapeutic benefits of exopeptidase combinations for patients with gluten-related health disorders including celiac disease.

## Introduction

Celiac disease (CeD) is a common autoimmune-like enteropathy affecting 1-1.4% of the global population (1, 2) with its incidence rising by 7.5% annually over the past several decades (3). CeD is caused by an aberrant response to specific peptide fragments released during dietary gluten digestion. A limited number of gluten immunogenic peptides (GIPs) are considered to be immunodominant, including the α_2_-gliadin derived 33-mer peptide which carries six overlapping T cell epitopes (4). The 33-mer is thought to be stable to proteolytic digestion by human gastric, pancreatic, and intestinal peptidases due to its abundance of proline residues (5), though studies of duodenal biopsies have shown the 33-mer to be almost fully degraded during intestinal transport in healthy individuals (6). Interestingly, the 33-mer peptide has also been shown to spontaneously form peptide self-aggregates *in vitro* (7, 8), which may further interfere with its digestion. In patients with CeD, GIP exposure reactivates a CD4+ T cell-driven immunological response resulting in a broad range of gastrointestinal (GI) and systemic symptoms (as reviewed elsewhere (9, 10)). A lifelong gluten-free diet (GFD) is seen as the only effective approach to prevent gastrointestinal symptoms in CeD patients. However, most patients do not experience complete mucosal healing on a GFD even with well-controlled symptoms (11, 12) and up to 80% of GFD-adhering patients experience inadvertent gluten contamination (13), highlighting a need for more effective CeD therapeutics.

Enzyme therapies currently in development aim to support the body’s natural digestion of gluten peptides using exogenous bacterial, fungal, or plant-derived peptidases. These therapies have almost exclusively focused on stomach-acting endopeptidases (ENPs), a class of enzyme that generates peptides of variable lengths by cleaving intra-chain residues (e.g., AN-PEP, Latiglutenase, TAK-062). While ENPs, such as pepsin, trypsin, and chymotrypsin, partially digest peptides into progressively smaller chains, exopeptidases (EXPs) complete digestion by systematically cleaving peptide bonds on either terminal end into absorbable lengths (*e.g.*, single amino acids, dipeptides). The majority of EXPs are anchored to enterocytes at the brush border membrane (BBM) or released by BBM vesicles into the lumen of the small intestine (14, 15). Patients with CeD experience damage to the intestinal BBM and reduced activity of certain brush border EXPs (16, 17). For instance, activity of the endogenous proline-specific dipeptidyl peptidase-IV (DPP-IV) was shown to be reduced by an average of 70% in CeD patients compared to healthy individuals without gastrointestinal diseases (17), likely aggravating the indigestibility of GIPs and their accumulation.

Here, we investigated a novel enzyme therapy approach for the digestion of GIPs using a combination of two exopeptidases, AMYNOPEP, that complements the intrinsic exopeptidase activity of enterocytes. AMYNOPEP consists of two tandem-acting aminopeptidases (a monoaminopeptidase and dipeptidyl peptidase) that digest peptides from the amino- to carboxy-terminal to generate absorbable single amino acids and dipeptides. We assessed the action of AMYNOPEP enzymes on stable isotope (SI) labelled 33-mer peptide digestion using a quantitative LC-MS/MS method for near-real-time detection of SI-labelled amino acids in the blood of healthy volunteers.

## Results

### AMYNOPEP rapidly and completely degrades the 33-mer peptide into dipeptides and single amino acids *in vitro*

**Figure 1:**
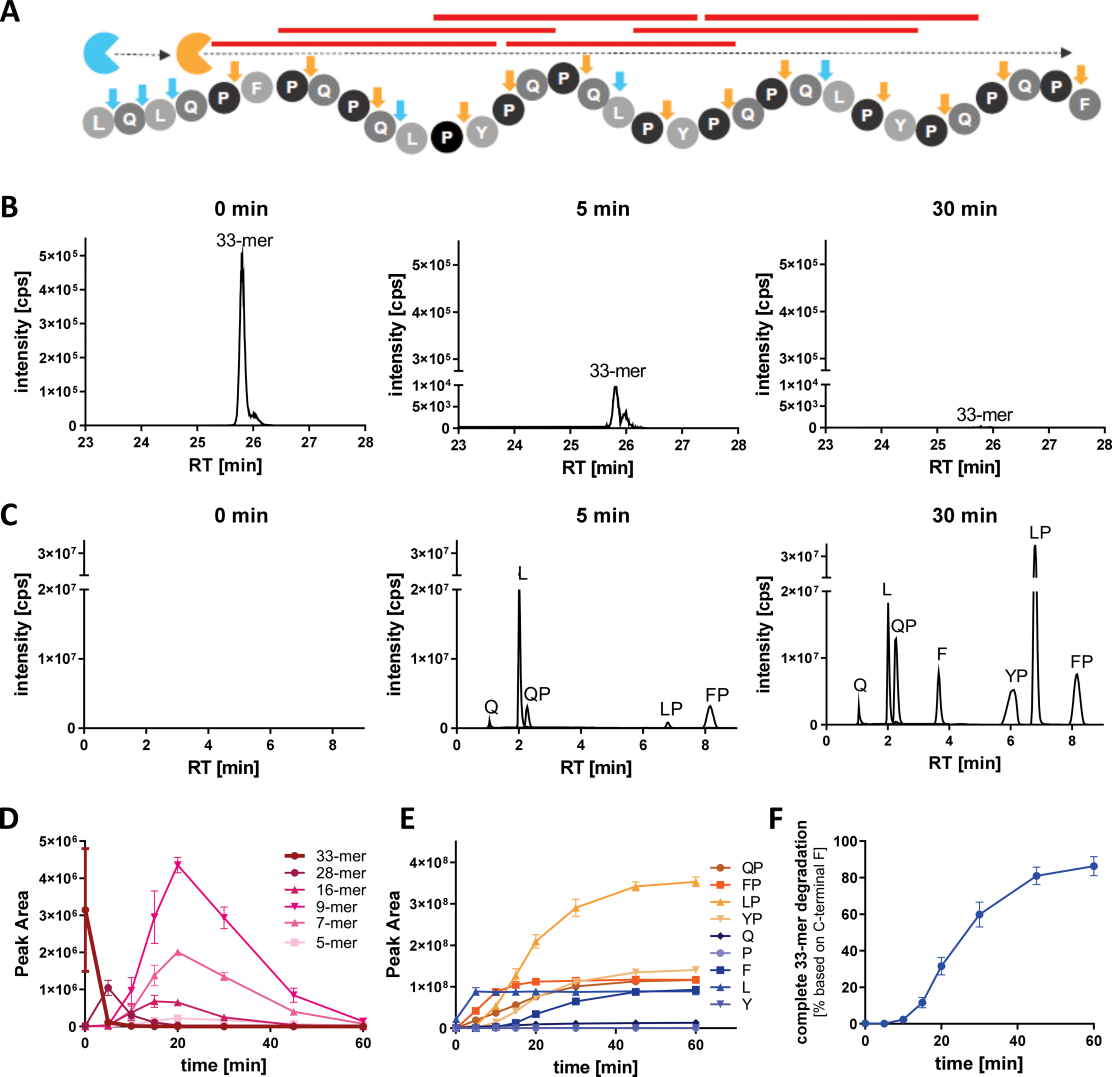
AMYNOPEP rapidly and completely degrades the 33-mer GIP into single amino acids and dipeptides *in vitro.* **(A)** Schematic representation of the AMYNOPEP mode-of-action. Tandem EXP activity ensures systematic degradation of the 33-mer through stepwise cleavage of single amino acids (L, Q, F), mediated by the aminopeptidase (blue), and cleavage of XP dipeptides (QP, FP, LP, YP) mediated by the dipeptidyl peptidase (orange). Cleaving sites for each enzyme are indicated by arrows. Red lines represent immunogenic HLA-DQ2/-DQ8 epitopes. **(B)** LC-MS/MS analysis of full length 33-mer degradation 0, 5, and 30 min after addition of AMYNOPEP. **(C)** Detection of XP dipeptides and amino acids during 33-mer degradation 0, 5, and 30 min after addition of AMYNOPEP. Peaks are labelled with their corresponding degradation product. **(D)** Time-dependent degradation of the 33-mer by AMYNOPEP and appearance of selected peptide intermediates, 28-mer (FPQPQLPYPQPQLPYPQPQLPYPQPQPF), 16-mer (LPYPQPQLPYPQPQPF), 9-mer (LPYPQPQPF), and 5-mer (QPQPF). **(E)** Corresponding time-dependent appearance of XP dipeptides and free amino acids. **(F)** Quantitative determination of complete 33-mer degradation based on the detection of F that is only cleaved once as single amino acid during the last cleavage step. **(D–F)** show means and SD of three technical replicates.

The degradation activity and efficacy of AMYNOPEP was assessed on the 33-mer *in vitro* (1:10 enzyme ratio, see **Methods**). Since both enzymes are aminopeptidases, the 33-mer was expected to be degraded through stepwise cleaving events beginning at the amino-terminal end and resulting in the release of single amino acids (leucine, L; glutamine, Q; phenylalanine, F) and X-proline (XP) dipeptides (QP, FP, LP, and (tyrosine, Y) YP, **Figure 1A**). Within 30 minutes of incubation with AMYNOPEP, less than 0.05% of the full 33-mer sequence was detectable (**Figure 1B**), and all anticipated degradation products (XP dipeptides and single amino acids) were detected (**Figure 1C**). We further analyzed the extent of 33-mer digestion by monitoring the appearance of degradation intermediates: 28-mer, 16-mer, 9-mer, and 5-mer peptides (**Figure 1D**). The degradation intermediates appeared in a time-dependent manner, in line with progressive N-to-C terminal degradation of 33-mer peptides. Accordingly, XP dipeptides and free amino acids increased in a time-dependent manner (**Figure 1E**). XP dipeptides appeared to be stable to enzymatic cleavage, as indicated by the lack of detectable free P or Y residues. Given the lack of XP cleavage, detection of free F residues indicated complete degradation of the 33-mer down to the carboxy-terminal-most F residue. Thus, the presence of detectable F within 30 minutes of AMYNOPEP incubation proved complete N-to-C terminal degradation of the 33-mer peptide *in vitro*. Using the terminal F, it could be estimated that 81% to 91% of analyzed 33-mer was degraded completely down to single amino acids and XP dipeptides after 60 minutes of incubation (**Figure 1F** **).** To further assess the complete degradation of the 33-mer peptide by AMYNOPEP *in vitro*, we monitored all theoretically possible 33-mer degradation intermediates expected to result from AMYNOPEP’s mode-of-action, revealing complete degradation of the measured peptide products by 60 minutes (**Supplementary Figure 1**). Faster 33-mer degradation *in vitro* was observed with an enzyme ratio of 1:1 (**Supplementary Figure 2**), thus a 1:1 enzyme ratio was used for all subsequent studies in healthy volunteers.

### SI-labelled amino acids are detectable in the plasma of healthy volunteers

**Figure 2:**
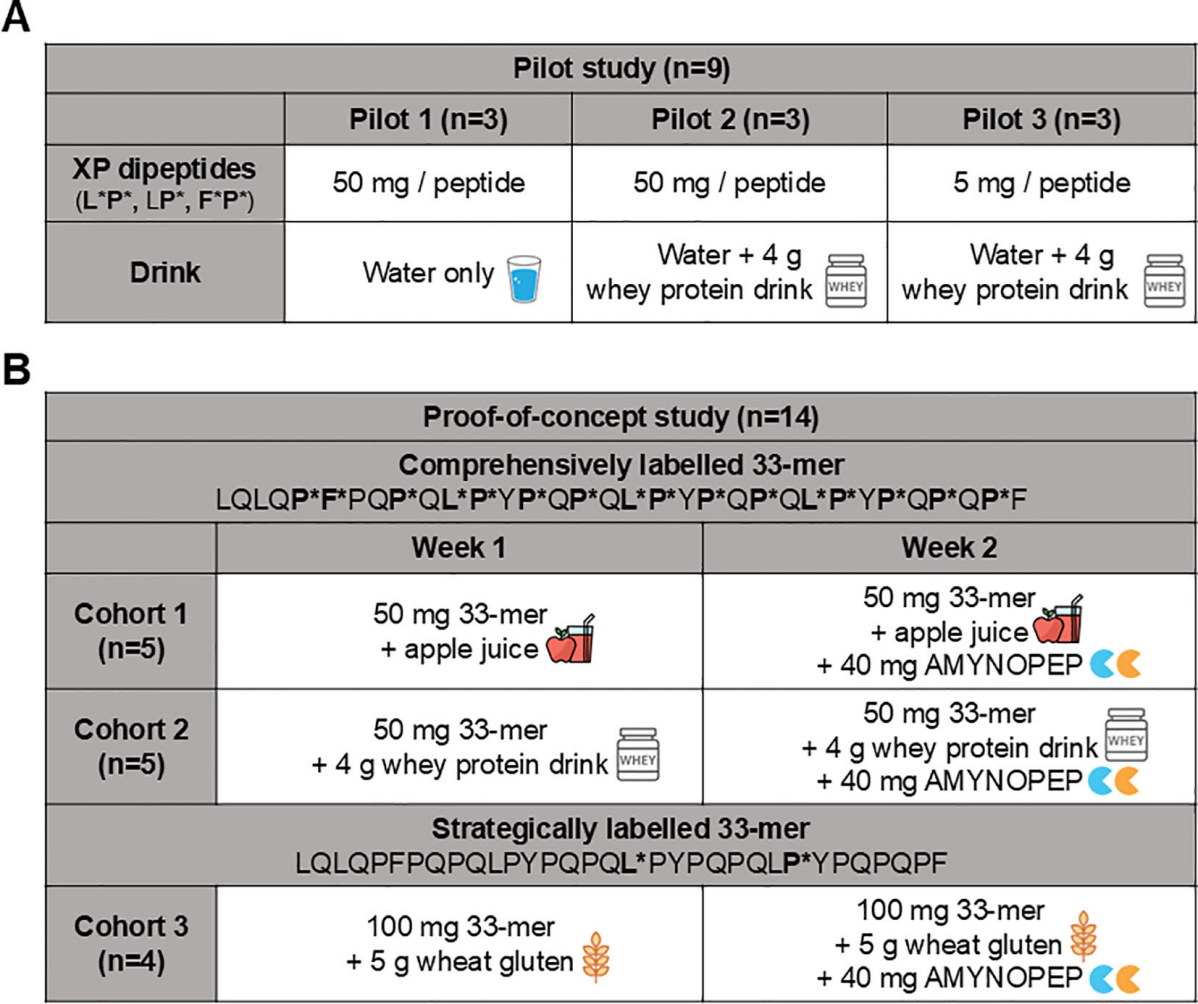
Overview of pilot and proof-of-concept study design assessing AMYNOPEP supplementation on 33-mer peptide digestion in healthy volunteers. *Pilot study:* To assess detection of orally administered amino acids in blood, a pilot study was conducted beforehand. **(A)** SI-labelled XP dipeptides were administered and resorption in the form of SI-labelled amino acids was measured in blood. **(B)** *Proof-of-concept study (Cohorts 1-3):* Two different isotope labelled peptides were used to evaluate 33-mer degradation: (1) a comprehensively labelled peptide with labels covering epitopes throughout the peptide, and (2) a strategically labelled peptide to evaluate end-to-end peptide digestion. A cross-over study design was utilized in which healthy volunteers followed a specified protocol on week one, followed by the same protocol with the addition of AMYNOPEP on week 2. In cohort 1, five volunteers were given 50 mg of comprehensively labelled peptide with apple juice. In cohort 2, five volunteers were given 50 mg of comprehensively labelled peptide with Peptamen. In cohort 3, four volunteers were given 100 mg of strategically labelled peptide with 5 g of wheat gluten. Blood was collected at regular intervals and 33-mer digestion products were measured as described in the Methods.

**Figure 3:**
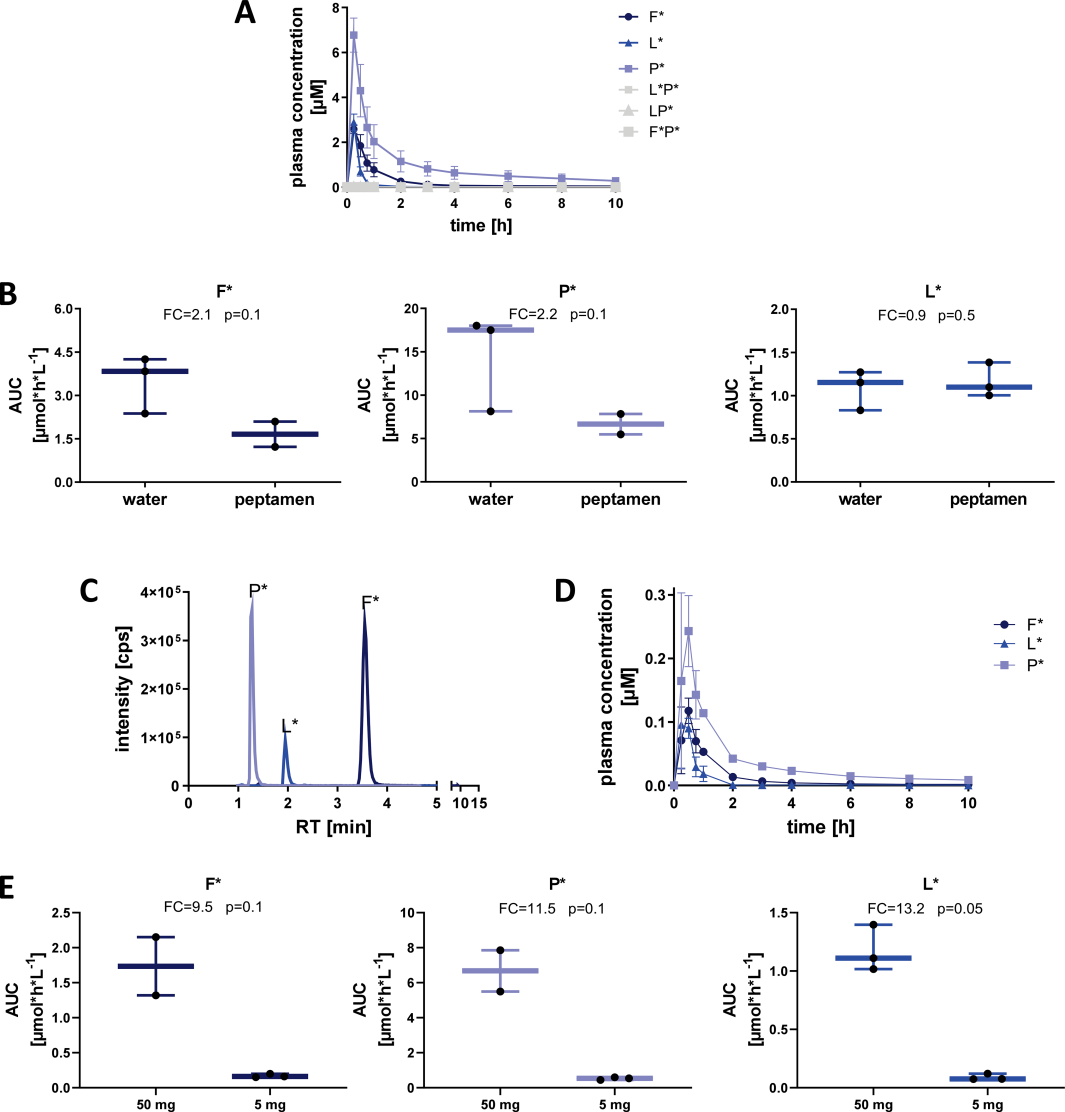
Detection of SI-labelled single amino acids in plasma after oral administration of XP dipeptides in healthy volunteers. **(A)** Plasma concentration [µM] of labelled dipeptides and amino acids after oral administration of L*P*, LP*, and F*P* (50 mg each) with water. Means and SD of three individuals are shown. **(B)** Influence of Peptamen (hydrolyzed protein) on absorption and detection of labelled amino acids after oral administration of dipeptides (50 mg each). Means and min/max of three individuals are shown. **(C)** LC-MS/MS detection intensity [cps] of labelled amino acids 1 hour after oral administration of dipeptides (5 mg each) taken with Peptamen. **(D)** Plasma concentration [µM] of labelled amino acids after administration of 5 mg of each labelled XP taken with Peptamen. **(E)** Influence of dose reduction from 50 mg to 5 mg of each labelled XP taken with Peptamen on plasma AUC of labelled amino acids. Box plots show the median, 25^th^ to 75^th^ percentiles and whisker show the min/max of three healthy individuals per cohort. FC, fold-change; AUC, area under the curve.

To evaluate the *in vivo* detection efficacy of labelled 33-mer degradation products, we performed a pilot study wherein SI-labelled XP dipeptides were orally administered to healthy volunteers (n=9 total) and subsequently measured in plasma and urine using HPLC-MS/MS (**Figure 2**, top panel pilot cohort). Volunteers (n=3) were given 50 mg of each SI-labelled XP dipeptide (F*P*, LP*, and L*P*) diluted in water, and the appearance of SI-labelled products was assessed over 72 hours in plasma and pooled urine samples. Only trace amounts (<LOQ) of XP dipeptides were detected in plasma (**Figure 3A**) and urine. In contrast, all three SI-labelled amino acids (L*, F*, and P*) that would result from the cleavage of their respective XP dipeptides were detectable in plasma in a time-dependent manner following oral administration (**Figure 3A**). Since SI-labelled amino acids were only partially excreted in urine with 0.002% to 0.017% of the orally administered amount (data not shown), subsequent *in vivo* studies did not include urine analyses.

To assess differences in SI-labelled amino acid digestion and absorption in the presence of a caloric load and competing peptide substrate, we administered 50 mg of each XP dipeptide with 100 mL of Peptamen (n=3 volunteers, **Figure 2** top panel). Peptamen did not influence the detection of XP dipeptides which were again only present in trace amounts in the plasma (data not shown). Peptamen resulted in decreased detection of F* and P* in plasma, as seen through a decrease in AUC of 2.1-fold and 2.4-fold, respectively (**Figure 3B**). In contrast, detection of L* was not significantly altered in the presence of Peptamen relative to water.

To assess the detection limit of our system, we lowered the dipeptide dose to 5 mg per XP, in combination with 100 mL Peptamen (n=3 volunteers, **Figure 2** top panel). The 10-fold dipeptide dose reduction resulted in clearly detectable F*, P*, and L* in plasma (**Figures 3C, D**), with a proportional reduction in plasma AUC of 9.5-fold, 11.5-fold, and 13.2-fold, respectively (**Figure 3E**). Based on the XP dipeptide absorption data, we proceeded to administer at least 50 mg of labelled 33-mer peptides in subsequent studies.

### AMYNOPEP supplementation rapidly increases the degradation of 33-mer peptides in healthy volunteers

**Figure 4:**
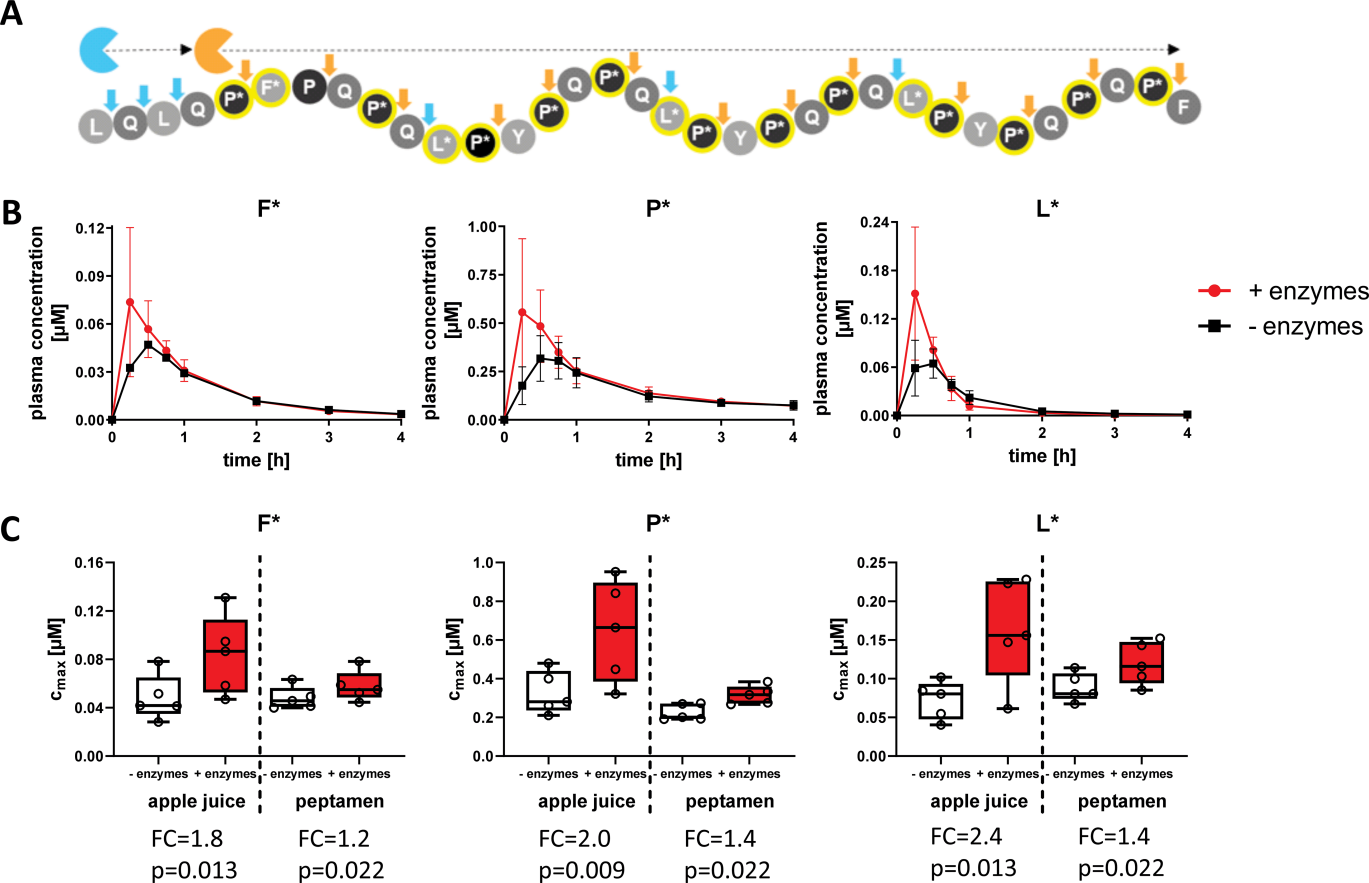
Rapid and enhanced degradation of the 33-mer peptide with AMYNOPEP supplementation. **(A)** Schematic overview of comprehensively labelled 33-mer peptide digestion by AMYNOPEP enzymes (represented by blue and orange sectors). Cleavage sites are indicated by arrows. **(B)** 50 mg of labelled 33-mer peptide was administered to five healthy volunteers with apple juice, preceded by administration of AMYNOPEP (visit 2) or plain water (visit 1). Plasma concentration of labelled amino acids (F*, P*, L*) was measured over the course of four hours. Means and SD of five healthy individuals are shown **(C)** C_max_ of labelled amino acids in plasma, with and without AMYNOPEP supplementation, and co-administration of apple juice (left side of graphs) or Peptamen (right side of graphs). Box plots show the median, 25^th^ to 75^th^ percentiles and whisker show the min/max of five healthy individuals. FC, fold-change; C_max_, maximum concentration.

To assess enzyme efficacy in healthy volunteers, we analyzed degradation of two different SI-labelled 33-mer peptides with and without AMYNOPEP (**Figure 2**, middle and lower panels). The first was a comprehensively labelled peptide with SI labels distributed throughout the sequence, covering all immunogenic epitopes with the goal of ensuring sufficient detection of enzyme activity *in vivo* (**Figure 4A**). In the first cohort (n=5 volunteers, **Figure 2** middle panel), 50 mg of comprehensively labelled 33-mer peptide were administered with apple juice, a low-caloric fluid to facilitate rapid gastric emptying, and to avoid the introduction of competing peptides that could affect enzyme activity. Interestingly, 33-mer degradation was also observed in volunteers without AMYNOPEP, though AMYNOPEP supplementation resulted in significantly higher plasma C_max_ of all labelled amino acids in the first 30 min post-administration compared to control (**Figures 4B, C**). Specifically, AMYNOPEP resulted in significant 1.8, 2.0, and 2.4-fold-change increases in maximum plasma concentrations (C_max_) of F*, P*, and L*, respectively (**Figure 4C**). In addition, AMYNOPEP supplementation resulted in significantly increased AUCs of F* (FC=1.3, p=0.025), P* (FC=1.2, p=0.006) and L* (FC=1.3, p=0.018) compared to control (**Supplementary Figure 3**).

In the second cohort (n=5 volunteers, **Figure 2** middle panel), we assessed the effect of competing peptide substrates in the form of 100 mL Peptamen on the degradation activity of 33-mer by AMYNOPEP. As expected, the addition of Peptamen decreased the efficacy of 33-mer digestion when compared to enzyme administration with apple juice, as shown by decreased C_max_ (**Figure 4C**). However, even with Peptamen, significant increases in C_max_ of F* (FC=1.2, p=0.022), P* (FC=1.4, p=0.022) and L* (FC=1.4, p=0.022) were observed with AMYNOPEP compared to control. Additionally, AMYNOPEP supplementation still resulted in significantly increased AUCs of F* (FC=1.1, p=0.022), P* (FC=1.2, p=0.022) and L* (FC=1.1, p=0.022) in the presence of Peptamen compared to controls.

AMYNOPEP supplementation significantly decreased time to C_max_ (t_max_) for F* (t_max_= 22.5 min with AMYNOPEP vs 31.5 min without, p=0.048) and P* (t_max_= 24 min with AMYNOPEP vs. 33 min without, p=0.032) when administered with apple juice (**Supplementary Figure 4**). No significant differences in t_max_ were observed with Peptamen (**Supplementary Figure 4**).

**Figure 5:**
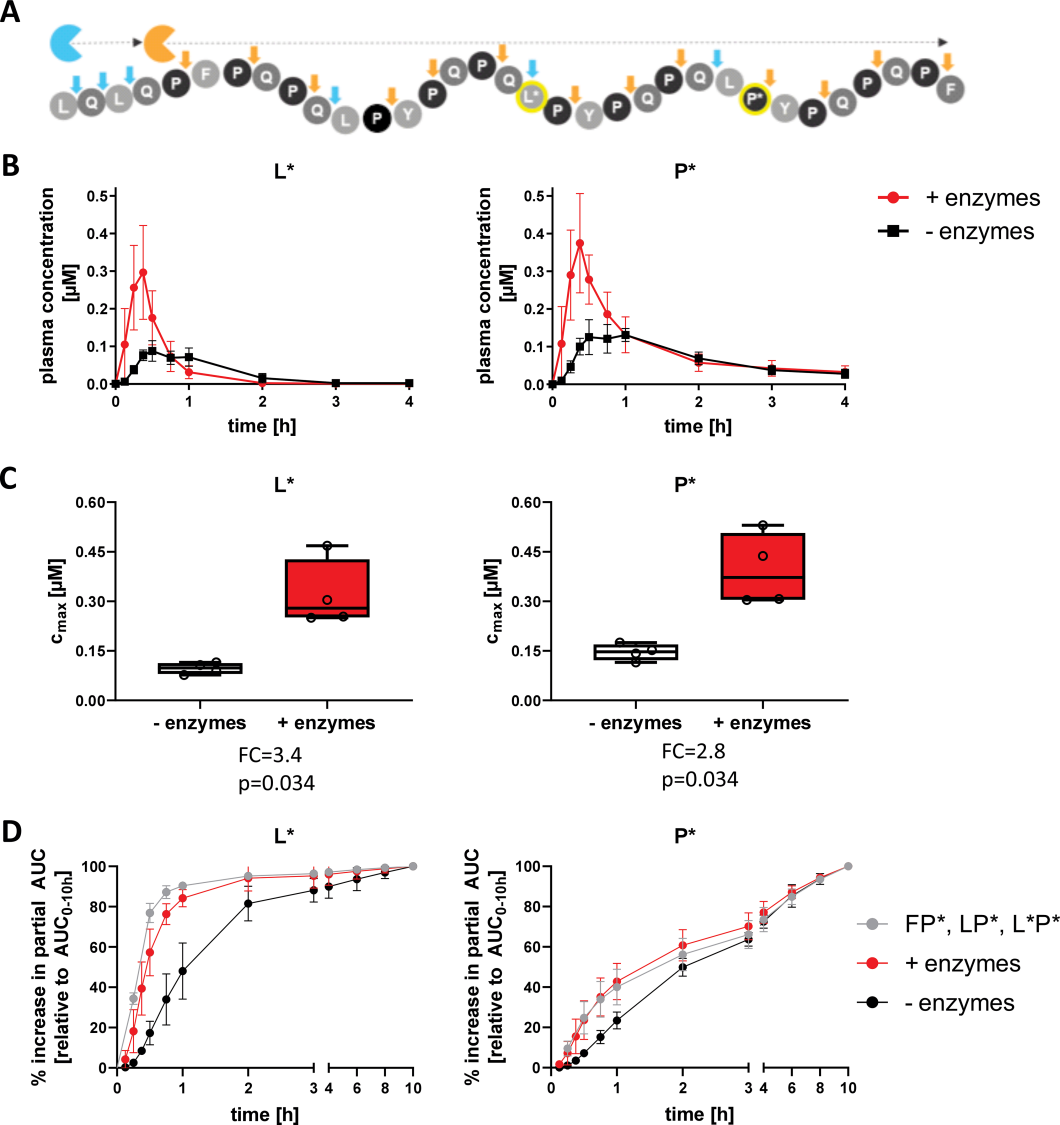
Comprehensive degradation of SI-labelled 33-mer by AMYNOPEP in the presence of gluten. **(A)** Schematic overview of strategically labelled 33-mer peptide digestion by AMYNOPEP enzymes (represented by blue and orange sectors). Cleavage sites are indicated by arrows. **(B, C)** 100 mg of strategically labelled 33-mer peptide was administered to four healthy volunteers with 5 g wheat gluten, with (red) or without (black) AMYNOPEP. Influence of enzyme addition on L* and P* plasma concentrations over time **(B)** and on maximum plasma concentrations (C_max_, **C)** were determined. **(D)** Comparison of amino acid absorption after administration of XP dipeptides (F*P*, LP*, L*P*) with water or 33-mer strategically labelled peptide with and without addition of AMYNOPEP. Plasma concentration-time plots show means and SD of four healthy individuals. Box plots show the median, 25^th^ to 75^th^ percentiles and whiskers show and min/max of four healthy individuals. C_max_, maximum concentration; AUC, area under the curve; FC, fold-change.

We further assessed AMYNOPEP enzyme efficacy using a second SI-labelled 33-mer peptide with strategic labeling of single L* and P* residues, to assess the extent of N-to-C terminal peptide digestion (**Figure 2** bottom panel, **Figure 5A**). Detection of L* would indicate degradation of the first half of the 33-mer and disruption of four out of six immunogenic epitopes, while the downstream P* shows disruption of all immunogenic epitopes. In a third cohort (n=4 volunteers), we administered 100 mg of the strategically labelled 33-mer with 5 g of wheat gluten as a competitive substrate (**Figure 2**). AMYNOPEP supplementation resulted in significantly increased C_max_ of P* (FC=2.8, p=0.034) and L* (FC=3.4, p=0.034) compared to control, even in the presence of gluten (**Figures 5B, C**). We observed a trend in which AMYNOPEP decreased t_max_, which was reached at 22.5 min post-administration of AMYNOPEP for both P* and L*, compared to t_max_ of 45 min without enzyme (p=0.054 for both; **Supplementary Figure 5**).

Most importantly, the absorption kinetics of single amino acids resolving from 33-mer degradation after AMYNOPEP addition closely resembled that of the amino acids when administered as XP dipeptides in the pilot study (**Figure 5D**). This finding reinforces the rapid action of AMYNOPEP, whereby the digestion and absorption of amino acids from a long (>30-amino acid) peptide was comparable to that of ingested dipeptides.

## Discussion

We have demonstrated rapid, systematic N-to-C terminal degradation of 33-mer peptides by AMYNOPEP *in vitro*, with corresponding degradation of SI-labelled 33-mer peptides in healthy volunteers even in the presence of competing substrates (Peptamen, wheat gluten). While the study was limited by its focus on one representative SI-labelled GIP, the positive results described herein warrant further investigation into AMYNOPEP’s efficacy at digesting other proteolytically-resistant and immunogenic peptides. Given the observed end-to-end degradation mode of action of AMYNOPEP *in vitro*, the same mechanism very likely underlies the improved degradation of 33-mer peptides *in vivo*, with AMYNOPEP supporting endogenous digestive enzyme machinery. Our strategy to use SI-labelled 33-mer combined with quantitative LC-MS/MS detection allowed us to distinguish between SI-labelled 33-mer degradation products and naturally occurring amino acids in plasma samples, including those from competing substrates co-administered with SI-labelled 33-mer. Considering the N-to-C terminal degradation of peptides by AMYNOPEP, the two differently SI-labelled peptides provided complementary information. Whereas the comprehensively labelled peptide allowed for sensitive detection of even minor degradation, the strategically labelled peptide gave us an indication of the extent of 33-mer digestion. Following the mode-of-action demonstrated *in vitro*, detection of labelled L* would indicate degradation of half of the 33-mer and four out of six immunogenic epitopes, whereas detection of labelled P* would show further N-to-C terminal degradation and disruption of all six immunogenic epitopes.

We observed 33-mer degradation in healthy volunteers even without AMYNOPEP. These findings further support that the 33-mer is not indigestible in healthy humans, but that its digestion in the small intestine may be performed by rate-limited enzymes (*e.g.*, brush border EXPs (18)). In line with this, research on duodenal biopsies from active CeD, GFD-managed CeD, and healthy volunteers showed that enterocytes of healthy individuals and GFD-managed, but not active CeD patients, can fully digest certain digestion-resistant gliadin peptides (6), suggesting defects in BBM peptidase activity in active CeD. As noted, compromised enterocyte functionality and reduced activity of certain BBM peptidases in CeD reinforce the need to supplement or replace BBM deficiencies. To date, there have been limited research and clinical studies aimed at developing EXP-based enzyme therapies, perhaps due to a limited understanding of the human BBM proteome and lack of suitable *in vitro* models to study the human BBM (19, 20), the source of most EXPs (14). Some exogenous EXPs have been studied in human and commercial settings, including DPP-IV and separately, leucyl aminopeptidase, demonstrating favorable safety profiles of these enzymes. DPP-IV is already available as an over-the-counter dietary supplement in several products, and a food enzyme leucyl aminopeptidase recently underwent safety evaluation showing no safety concerns under conditions of use in eight food manufacturing processes (21). Regarding the safety of AMYNOPEP, we collected data on adverse events during the course of study participation, and no volunteers reported adverse events related to the ingestion of study materials (AMYNOPEP or SI-labelled peptides, see **Appendix I** for more details on safety data). We demonstrated here that EXP supplementation *via* AMYNOPEP greatly increased 33-mer degradation and SI-labelled amino acid absorption *in vivo*, even in healthy volunteers with presumably intact BBM. Our approach, with quantitative LC-MS/MS measurement of SI-labelled 33-mer degradation products, allowed for sensitive, non-invasive monitoring of GIP degradation in near-real-time. We did not detect SI-labelled XP dipeptides *in vivo*, likely due to endogenous processing of dipeptides into single amino acids by enterocytes (as demonstrated in the pilot study).

Contrary to many ENP-based enzyme therapies under development for the treatment of CeD, our enzyme combination targets peptide degradation in the small intestine. The focus of most enzyme therapies on peptide digestion in the stomach likely stems from concerns that GIP digestion must occur before gastric emptying to prevent immune activation in the small intestine. However, recent research into the immunological timeline of events following gluten ingestion revealed a one-to-four-hour window before peak detection of immune markers in the blood and associated gastrointestinal symptoms (22), suggesting that fast-acting enteric enzymes may still digest gluten peptides in time to prevent adverse effects. Furthermore, enzyme approaches relying solely on gluten digestion in the stomach are likely to be ineffective due to limited mixing in the stomach (23, 24) and rapid gastric emptying of low-caloric fluids and small particles (23, 25). Stomach-targeting enzyme supplements may therefore be rapidly emptied into the small intestine, where they might experience greatly reduced activity or inactivation due to changes in pH or vulnerability to trypsin, thereby missing their window of opportunity to efficiently digest gluten peptides. Our novel mode-of-action is based on combinatorial EXP action in the small intestine, hence addressing these limitations, complementing the body’s EXP activity of luminal or brush border origin to achieve thorough digestion of GIPs into absorbable single amino acids and dipeptides. While our study was limited by a small sample size of healthy volunteers and did not include patients with CeD, our SI-labelled approach offers a highly sensitive and accurate detection method for assessing enzyme efficacy in near real-time. Future enzymatic approaches to gluten digestion should account for issues of motility and enzyme mode-of-action, with a particular focus on intestinal activity and EXP supplementation.

## Materials and methods

### Materials

All labelled peptides, XP dipeptides, and amino acids were synthesized using 13C and 15N stable isotopes (SI, labelling further indicates as *). Labelled 33-mer (LQLQP*F*PQP*QL*P*YP*QP*QL*P*YP*QP*QL*P*YP*QP*QP*F and LQLQPFPQPQLPYPQPQL*PYPQPQLP*YPQPQPF) and XP dipeptides phenylalanine-proline (F*P*), leucine-proline (LP*), and leucine-proline (L*P*) were synthesized by Intavis Peptide Services GmbH (Tübingen, Germany). Labelled and non-labelled amino acids (F, P, L, Y, Q) were obtained from Sigma-Aldrich (Taufkirchen, Germany). Non-labelled 33-mer was obtained from Ontores biotechnologies (Shanghai, China). Non-labelled XP dipeptides FP, LP, YP and QP as well as 28-mer, 16-mer, 9-mer and 5-mer were obtained as SpikeTides™ for method development from JPT Peptide Technology GmbH (Berlin, Germany). LC-MS/MS grade acetonitrile, methanol, and formic acid were obtained from Merck (Darmstad, Germany). LoBind 1.5mL reaction tubes were obtained from Sarstedt (Nümbrecht, Germany). Peptamen™ (Neutral SmartFlex) was obtained from Nestlé Health Science Gmbh (Frankfurt, Germany). Wheat gluten was obtained from Veganz Group AG (Berlin, Germany). Hydrogencarbonate (NaHCO_3_) was obtained from Arnold Holste Wwe. GmbH & Co. KG (Bielefeld, Germany).

### *In vitro* digestion of 33-mer peptide by AMYNOPEP

*In vitro* digestion of 33-mer peptides was performed using a two-form enzyme combination, AMYNOPEP, which utilizes two tandem-acting aminopeptidases. The 33-mer digestion was done in Tris buffer at pH 7.0 using an enzyme mixture consisting of 0.86 mg/L dipeptidyl peptidase and 8.6 mg/L aminopeptidase (1:10 enzyme ratio). Enzyme mixture was incubated with 100 mg/L 33-mer in a final volume of 1.8 mL. Digestions were performed at 37°C and 350 rpm. Samples of 200 µL were collected at 0, 5, and 30 min and immediately transferred to new 1.5 mL reaction tubes prefilled with 1.6 mL 0.1% TFA to stop the reaction. Samples were stored at -80°C prior to LC-MS/MS measurement, for which samples were diluted 1:5 in 0.1% formic acid. 5 µL or 10 µL of samples were analyzed to detect the intact 33-mer, degradation intermediates (28-mer: FPQPQLPYPQPQLPYPQPQLPYPQPQPF; 16-mer: LPYPQPQLPYPQPQPF; 9-mer: LPYPQPQPF; 5-mer: QPQPF), and degradation products (dipeptides and amino acids). In order to determine the proportion of complete degradation, the amount of cleaved F, which is only generated in the last step of degradation, was quantified and set in relation to the theoretical maximum achievable concentration with complete degradation of all 33-mer molecules. Detailed description of LC-MS/MS method to detect *in vitro* degradation products can be found in **Supplementary Tables 1**, **2**.

### Pilot study demonstrating SI-labelled amino acid detection in plasma of healthy volunteers

Healthy volunteers were selected based on no previous history of CeD, NCGS, or wheat allergy. The study (German Register of Clinical Studies, DRKS00033099) was approved by the Ethics Committee of the University Medicine Greifswald, and was performed in adherence to the declaration of Helsinki (2013 Version), the Medical Association’s Professional code of conduct for Mecklenburg-Vorpommern (BOÄ M-V) and the data protection regulation of the EU (EU-DSGVO) and of Mecklenburg-Vorpommern (DSG-MV). Written informed consent was obtained from all volunteers prior to enrollment in the study.

A mixture of all three SI labelled XP dipeptides (F*P*, LP*, and L*P*) were orally administered to volunteers (n=9 total), divided into three cohorts of three volunteers each (see **Figure 2**). Cohort 1 received 50 mg of each dipeptide in 300 mL water. Cohort 2 received 100 mL Peptamen first, then 50 mg of each dipeptide in 150 mL water, followed by another 50 mL water. All drinks were taken within a minute. Cohort 3 received 100 mL Peptamen first, then a reduced dose of 5 mg of each dipeptide in 150 mL water, followed by another 50 mL water. All drinks were taken within a minute. Blood samples were collected after 15 min, 30 min, 45 min, 1 h, 2 h, 3 h, 4 h, 6 h, 8 h, 10 h, 14 h, 48 h and 72 h after dosing. Urine was collected and pooled at 0-4 h, 4-6 h, and 6-10 h, and was collected as spot urine at 48 h and 72 h after dosing.

### Proof-of-concept study evaluating 33-mer peptide degradation by AMYNOPEP in healthy volunteers

Healthy volunteers were selected based on no previous history of CeD, NCGS, or wheat allergy. The study (German Register of Clinical Studies, DRKS00033108) was approved by the Ethics Committee of the University Medicine Greifswald, and was performed in adherence to the declaration of Helsinki (2013 Version), the Medical Association’s Professional code of conduct for Mecklenburg-Vorpommern (BOÄ M-V) and the data protection regulation of the EU (EU-DSGVO) and of Mecklenburg-Vorpommern (DSG-MV). Written informed consent was obtained from all volunteers prior to enrollment in the study.

Volunteers were divided into three cohorts, utilizing differently labelled 33-mer peptides and different food intake conditions. Two differently SI-labelled 33-mer peptides were used: (1) a comprehensively labelled 33-mer (LQLQP*F*PQP*QL*P*YP*QP*QL*P*YP*QP*QL*P*YP*QP*QP*F), and (2) a strategically labelled 33-mer (LQLQPFPQPQLPYPQPQL*PYPQPQLP*YPQPQPF). Bold asterisked letters indicate SI-labelled amino acids. The study was designed as a cross-over study, with study visits one week apart. During visit 1, each volunteer received the labelled 33-mer with apple juice, Peptamen, or wheat gluten without AMYNOPEP. During visit 2, each volunteer received the labelled 33-mer with apple juice, Peptamen, or wheat gluten plus AMYNOPEP. Consistently across the study cohorts, AMYNOPEP was prepared in a 1:1 enzyme ratio (20 mg of each enzyme) dissolved in 40 mL water with 500 mg sodium bicarbonate to neutralize gastric acid.

In cohort 1, volunteers (n=5) received 40 mL water with 500 mg sodium bicarbonate, and 5 min later, 50 mg of comprehensively labelled 33-mer dissolved in 20 mL water and 180 mL apple juice (week 1). Apple juice was treated with 2 g sodium bicarbonate before administration to neutralize fruit acids. In week 2, volunteers first received 40 mg of AMYNOPEP in 40 mL water with 500 mg sodium bicarbonate, and 5 min later, received the same treatment conditions as in week 1. In cohort 2, volunteers (n=5) followed the same study protocol as in cohort 1, but apple juice was replaced with 100 mL of Peptamen (containing partially hydrolyzed whey protein, 100 kCal, 4 g peptides per 100 mL; caloric content approximately similar to the volume of apple juice in cohort 1) neutralized with 1 g sodium bicarbonate. In cohort 3, volunteers (n=4) received 100 mg of strategically labelled 33-mer together with 5 g of wheat gluten dissolved in 180 mL of water (week 1). Wheat gluten was previously neutralized by 2 g of sodium bicarbonate. In week 2, volunteers received 40 mg of AMYNOPEP, and after 5 min, received the same treatment conditions as in week 1.

Blood samples were collected after 15 min, 30 min, 45 min, 1 h, 2 h, 3 h, 4 h, 6 h, 8 h, 10 h, and 24 h after treatments, and plasma amino acid levels were monitored at each time point by LC-MS/MS. In cohorts 2 and 3, additional blood samples were collected at 7.5 min and 22.5 min after treatments.

### Sample preparation

Plasma samples were thawed on ice, mixed, and 100 µL of each sample was transferred to a 1.5 mL LoBind reaction tube. Plasma samples were precipitated after adding 200 µL of acetonitrile/methanol (10:1), and incubated on ice for 15 min. Samples were centrifuged at 16,000 *g* for 15 min at 4 °C. 100 µL supernatant was dried under nitrogen at 40 °C. Samples were reconstituted in 100 µL 0.1% formic acid and 5 µL was applied to the LC-MS/MS instrument.

### Analysis of LC-MS/MS data

Labelled XP dipeptides and single amino acids (P*, L*, F*) in plasma samples were quantified by LC-MS/MS using Nexera LC40 HPLC system (Shimadzu, Duisburg, Germany) coupled to an API 6500+ ™ tandem mass spectrometer (AB Sciex, Darmstadt, Germany). For chromatographic separation an ACQUITY UPLC HSS PFP Column (100 Å, 1.8 µm, 2.1 mm X 150 mm; Waters, Eschborn, Germany) protected by a SecurityGuard C18 column (4x2 mm; Phenomenex, Aschaffenburg, Germany) was used. Chromatographic separation was performed at an oven temperature of 40°C and the solvent flow rate was set to 400 µL/min. Elution was achieved by varying composition of solvent A (90% acetonitrile + methanol (6 + 1), 0.1% formic acid in H_2_O) and solvent B (0.1% formic acid in H_2_O) using following LC conditions: 0-1.4 min 0% solvent A; 1.4-10.4 min 0-50% solvent A; 10.5-12.9 min 80% solvent A; 13-15 min 0% solvent A. To decrease the introduction of debris into the MS, a valve was directed to waste at 0.1-1 min and 9.5-14.9 min.

MS detection of labelled amino acids and XP dipeptides was performed in positive mode with curtain gas set to 40, gas 1 of 50, gas 2 of 70, temperature of 500°C and an IS of 3500. Detection was achieved using MS parameters and mass transitions listed in **Supplementary Table 3**.

The LC-MS/MS method was verified to be specific for the quantitative determination of P*, F*, L*, F*P*, LP* and L*P* in human plasma and urine. No interferences of the analytical signals with the biological matrix were observed. The calibration curves for labelled amino acids were linear between 0.002 µM and 5 µM for F*, P* and L*. This correlates to 0.24 to 605 ng/ml P*, 0.35 to 875 ng/ml F and 0.28 to 690 ng/ml L*. The calibration curves for F*P*, LP* and L*P* were linear between 0.005 µM and 5 µM. This correlates to 1.39 to 1390 ng/ml for F*P*, 1.17 to 1170 ng/ml for LP* and 1.21 to 1205 ng/ml for L*P*. The correlation coefficients for all analytes ranged between 0.9986 and 1.

Analysis of *in vitro* samples was performed semi quantitatively. Non-labelled XP dipeptides and single amino acids from *in vitro* samples were measured applying the method described above using mass transitions and MS parameters listed in **Supplementary Table 1**. To estimate total degradation, measurement of carboxy-terminal F that is only cleaved in the last step of 33-mer digestion by exopeptidases, was performed quantitatively. Detection of 33-mer and its degradation intermediates (28-mer, 16-mer, 9-mer, 7-mer and 5-mer; see **Supplementary Table 2**) was achieved using an ACQUITY UPLC BEH C18 Column (130Å, 1.7 µm, 2.1 mm X 50 mm (Waters) protected by a SecurityGuard C18 column (4x2 mm; Phenomenex, Aschaffenburg, Germany) for chromatographic separation. Chromatographic separation was performed at an oven temperature of 40°C and the solvent flow rate was set to 400 µL/min. Elution was achieved using following LC conditions: 0-4 min 0% solvent A; 4-6 min 0-15% solvent A; 6-20 min 15-30% solvent A; 20-26 min 30-40% solvent A, 26.1-28 min 60% solvent A, 28.1-31.2 min 0% solvent A. MS detection was performed in positive mode with curtain gas set to 40, gas 1 of 50, gas 2 of 50, temperature of 450°C and IS of 5500.

### Pharmacokinetics analyses

LC-MS/MS-generated chromatograms were analyzed using Analyst 1.7.2 (Sciex, Darmstadt, Germany). The pharmacokinetics parameter AUC was calculated using GraphPad Prism version 8.0 (GraphPad Prism Software Inc., La Jolla, CA, USA). Partial AUCs were calculated manually using the trapezoidal rule.

### Statistical analyses

Statistical analyses were performed using SPSS Statistics Version 28 (SPSS Inc., IBM, Chicago, IL, USA). To compare the effects between cohorts in the dipeptide resorption study the one-tailed Mann-Whitney-Test was applied. To compare the effects between enzyme supplementation and controls during the proof-of-concept study the one tailed Wilcoxon matched-pairs signed rank test was applied.

## Data Availability

The data generated during this study are available upon request from the corresponding authors. Access to the data will be considered on a case-by-case basis, and any requests will be evaluated in accordance with ethical guidelines and data sharing policies. Please contact the corresponding authors for further information.
