# Supplementary material for: Exopeptidase combination enhances the degradation of isotopically labelled gluten immunogenic peptides in humans
Source: Front Immunol. 2024 Oct 16;15:1425982. doi: 10.3389/fimmu.2024.1425982 (PMC11522800; doi:10.3389/fimmu.2024.1425982)
Supplement: Supplementary file 1 [file DataSheet1.pdf]

Supplementary Material

Supplementary Figures

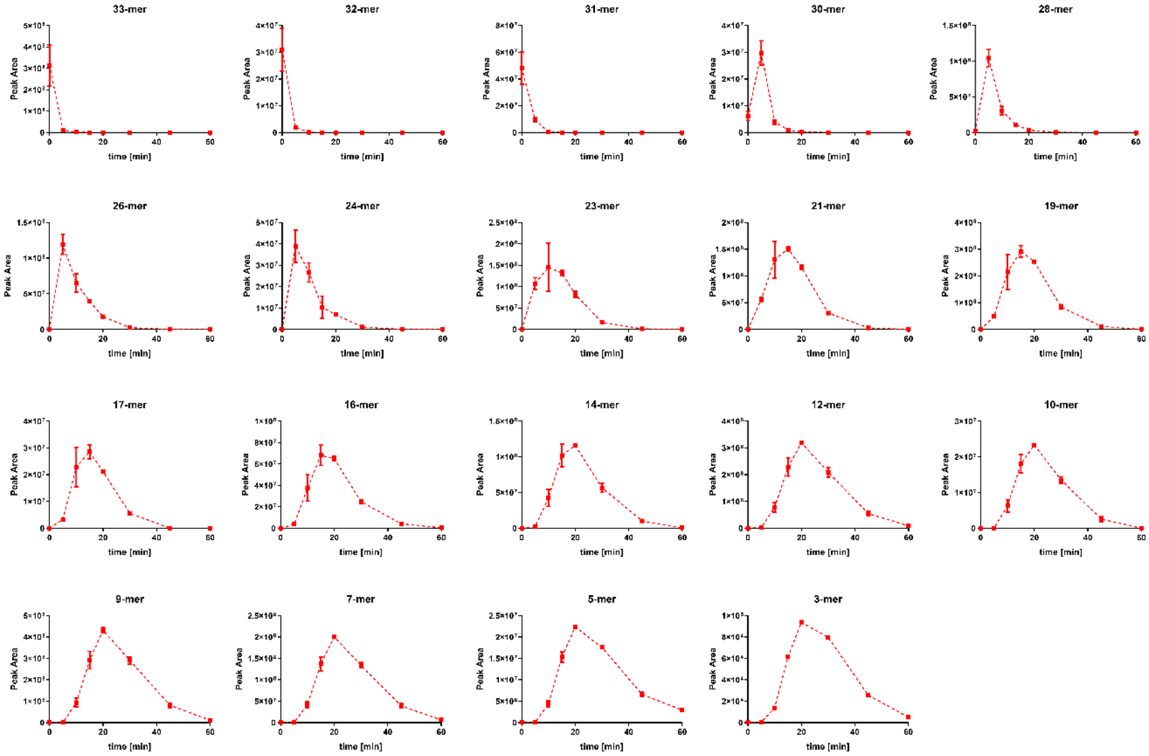

Supplementary Figure 1. *In vitro* degradation of 33-mer by AMYNOPEP and time-dependent decreases in resulting peptide fragments as resolved by LC-MS/MS. Monitoring of all theoretical 33-mer degradation products that result from AMYNOPEP digestion. Shown are means and SEM of three technical replicates.

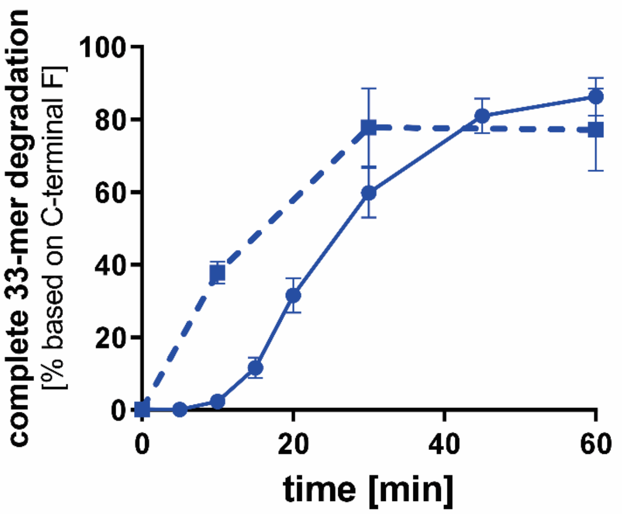

Supplementary Figure 2. Degradation of 33-mer *in vitro* with AMYNOPEP at an enzyme ratio of 1:1. Percentage of complete 33-mer peptide degradation (based on C-terminal F) after treatment with enzymes at 1:10 ratio dipeptidyl peptidase : aminopeptidase (solid line) or 1:1 ratio (dotted line). N= 3 biological replicates.

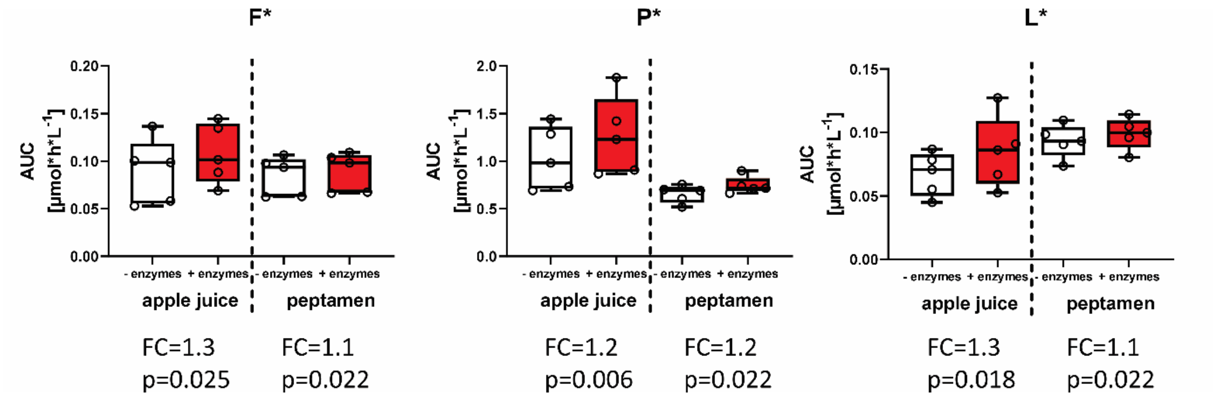

Supplementary Figure 3. Influence of AMYNOPEP on plasma AUC of labelled amino acids. Box plots show the median, 25th to 75th percentiles and whisker show and min/max of five healthy individuals per cohort. *Abbreviations:* FC=fold-change, AUC=area under the curve.

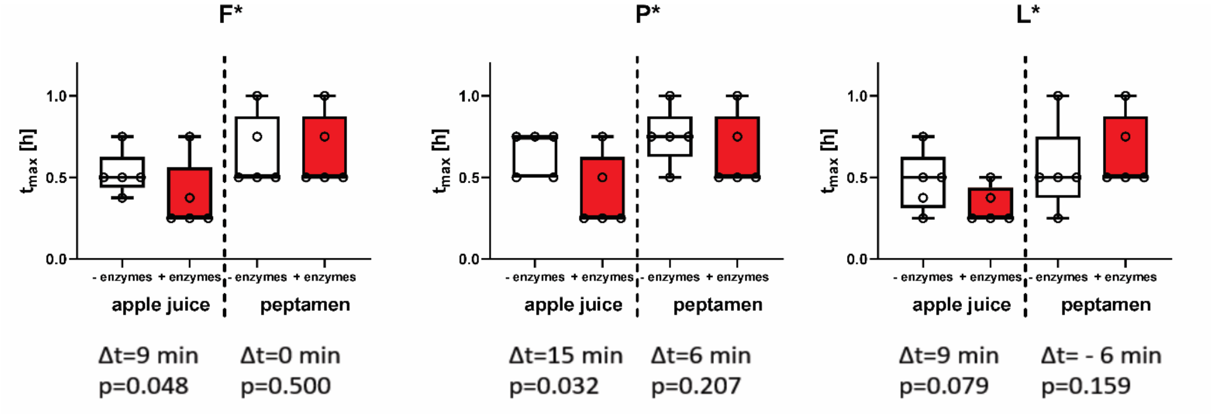

Supplementary Figure 4. Rapid degradation of 33-mer peptide with AMYNOPEP. 50 mg of labeled 33-mer peptide was administered to five healthy volunteers with apple juice or Peptamen, preceded by administration of AMYNOPEP (visit 2) or plain water (visit 1). Time to reach maximum plasma concentrations (t_{max}) is shown as box and whisker plots with median, 25th to 75th percentiles and min/max of five healthy individuals. Mean reduction in t_{max} is stated as Δt.

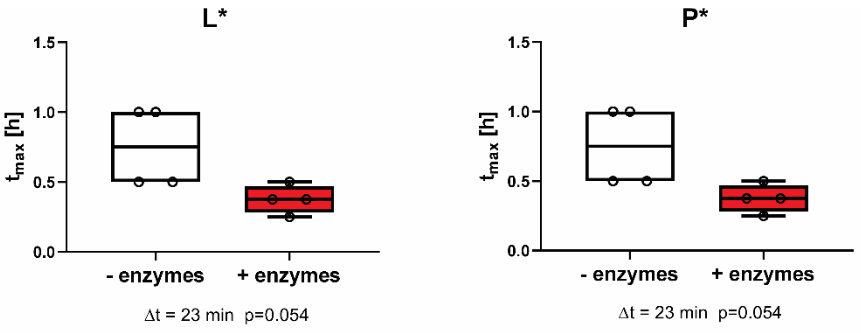

Supplementary Figure 5. Rapid degradation of 33-mer peptide with AMYNOPEP in the presence of gluten. 100 mg of labeled 33-mer peptide was administered to four healthy volunteers with 5g wheat gluten, proceeded by administration of AMYNOPEP (visit 2) or plain water (visit 1). Time till reaching maximum plasma concentrations (t_{max}) is shown as box and whisker plots with median, 25th to 75th percentiles and min/max of five healthy individuals. Mean reduction in t_{max} is stated as Δt.

Supplementary Tables

Analyte	Detected fragments (m/z)		DP	CE	EP	CXP
P	Quantifier:	116.1 > 70	40	22	11	8
F	Quantifier:	165.9 > 120.2	25	17	5	14
	Qualifier:	165.9 > 103	24	35	5	12
L	Quantifier:	132.2 > 86.2	28	14	12	9
	Qualifier:	132.2 > 44	23	30	11	6
Q	Quantifier:	147.0 > 84.1	24	24	12	10
	Qualifier:	147.0 > 56.1	24	40	12	9
Y	Quantifier:	182.1 > 136.2	23	19	11	8
	Qualifier:	182.1 > 123	20	23	10	14
QP	Quantifier:	244.1 > 116.1	35	25	9	14
FP	Quantifier:	263.1 > 116.1	37	20	9	10
	Qualifier:	263.1 > 120.1	37	30	9	14
LP	Quantifier:	229.1 > 116.1	33	18	7	14
	Qualifier:	229.1 > 86.1	32	22	9	11
YP	Quantifier:	279.1 > 116.1	40	20	9	12
	Qualifier:	279.1 > 136.2	50	25	9	11

Supplementary Table 1. MS parameters for detection of non-labeled XP dipeptides and single amino acids.

Analyte	Peptide Sequence	Detected fragments (m/z)		DP	CE	EP	CXP
33-mer	LQLQPFPQPQLPYPQPQLPYPQPQLPYPQPQPF	Transition 1:	978,7 > 263,2	90	34	12	10
		Transition 2:	978,7 > 488,4	90	33	12	19
32-mer	QLQPFPQPQLPYPQPQLPYPQPQLPYPQPQPF	Transition 1:	949,9 > 263,2	90	35	12	12
		Transition 2:	949,9 > 488,4	90	35	12	12
31-mer	LQPFPQPQLPYPQPQLPYPQPQLPYPQPQPF	Transition 1:	917,9 > 263,2	90	35	12	12
		Transition 2:	917,9 > 488,4	90	35	12	12
30-mer	QPFPQPQLPYPQPQLPYPQPQLPYPQPQPF	Transition 1:	1185,9 > 263,2	90	35	12	12
		Transition 2:	1185,9 > 488,4	90	35	12	12
28-mer	FPQPQLPYPQPQLPYPQPQLPYPQPQPF	Transition 1:	1111,0 > 263,2	130	41	11	17
		Transition 2:	1111,0 > 488,4	130	38	11	12
26-mer	QPQLPYPQPQLPYPQPQLPYPQPQPF	Transition 1:	1029,5 > 263,2	90	35	12	12
		Transition 2:	1029,5 > 488,4	90	35	12	12
24-mer	QLPYPQPQLPYPQPQLPYPQPQPF	Transition 1:	954,5 > 263,2	90	35	12	12
		Transition 2:	954,5 > 488,4	90	35	12	12
23-mer	LPYPQPQLPYPQPQLPYPQPQPF	Transition 1:	912,2 >263,2	90	30	10	13
		Transition 2:	912,2 > 488,4	90	26	10	13
21-mer	YPQPQLPYPQPQLPYPQPQPF	Transition 1:	842,0> >263,2	90	30	11	10
		Transition 2:	842,0 > 488,4	90	27	10	20
19-mer	QPQLPYPQPQLPYPQPQPF	Transition 1:	755,1 > 263,2	53	28	11	17
		Transition 2:	755,1 > 226,2	60	34	12	13
17-mer	QLPYPQPQLPYPQPQPF	Transition 1:	680,0 > 263,2	55	25	7	23
		Transition 2:	680,0 > 488,4	60	23	6	13
16-mer	LPYPQPQLPYPQPQPF	Transition 1:	955,6>263,2	90	37	10	13
		Transition 2:	955,6 > 488,4	90	35	10	13
14-mer	YPQPQLPYPQPQPF	Transition 1:	850,4 > 263,2	90	32	11	19
		Transition 2:	850,4 > 488,4	90	30	11	32
12-mer	QPQLPYPQPQPF	Transition 1:	720,4 > 263,2	55	28	8	16
		Transition 2:	720,4 > 488,4	62	24	11	32
10-mer	QLPYPQPQPF	Transition 1:	607,8 > 263,2	55	25	10	17
		Transition 2:	607,8 > 488,4	60	20	7	27
9-mer	LPYPQPQPF	Transition 1:	543,9 > 263,2	46	21	11	21
		Transition 2:	543,9 > 226,2	52	37	8	37
7-mer	YPQPQPF	Transition 1:	438,7 > 263,2	43	17	7	16
		Transition 2:	438,7 > 614,3*	43	14	7	16
5-mer	QPQPF	Transition 1:	616,3 > 166,1	100	36	7	15
		Transition 2:	616,3 > 263,2	100	33	7	17
3-mer	QPF	Transition 1:	390,7 > 166,0	42	21	8	15
		Transition 2:	390,7 > 207,9	50	30	8	14

Supplementary Table 2. MS parameters for detection of 33-mer and degradation intermediates that result from AMYNOPEP cleavage activity. Abbreviations: Declustering potential (DP), collision energy (CE), entry potential (EP), cell exit potential (CXP). * Transition equals mass/z of 877.4/+2 for the mother ion and 614.3/+1 of the daughter ion.

Analyte	Detected fragments (m/z)		DP	CE	EP	CXP
**P***	Quantifier:	122.0 > 75.0	30	23	11	12
**F***	Quantifier:	176.2 > 129.2	27	20	11	8
	Qualifier:	176.2 > 111.0	27	38	11	13
**L***	Quantifier:	139.2 > 92.1	20	11	15	5
**F*P***	Quantifier:	279.2 > 122.1	40	22	8	15
	Qualifier:	279.2 > 128.9	40	31	8	20
L**P***	Quantifier:	235.2 > 122.1	40	20	8	15
	Qualifier:	235.2 > 86.2	40	23	8	10
**L*P***	Quantifier:	242.3 > 122.1	40	19	8	14
	Qualifier:	242.3 > 92.1	40	22	8	14

Supplementary Table 3. MS parameters for detection of SI-labelled XP dipeptides and single amino acids. SI-labelled amino acids are indicated by **bold***.
